# P-2278. CMV Viremia and Disease in Pediatric Liver Transplant Patients: a Multi-national Systematic Review and Meta-analysis

**DOI:** 10.1093/ofid/ofae631.2431

**Published:** 2025-01-29

**Authors:** Mohamad Shieb, Eric J Stern, Rand Hasanain

**Affiliations:** medstar georgetown university hospital, ARLINGTON, Virginia; Medstar Georgetown Pediatrics, Washington, District of Columbia; Al Jalila Children’s Hospital, Twar, Dubai, United Arab Emirates

## Abstract

**Background:**

Cytomegalovirus (CMV) viremia (Defined as a single detectable CMV RNA PCR) and disease (defined as CMV viremia with compatible systemic and/or localized signs and symptoms) remain a leading cause for mortality and morbidity in the pediatric solid organ transplant recipient. This systemic review aims at pooling data from the leading pediatric transplant institutions globally to identify the overall incidence of and risk factors associated with CMV viremia and/or disease in pediatric liver transplant recipients.

PRISMA flow diagram

**Figure:**
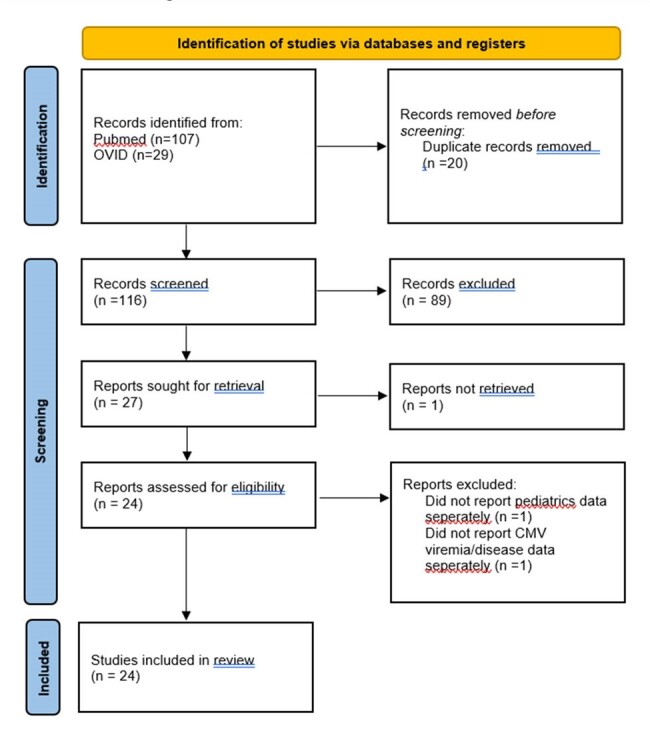

**Methods:**

A systemic review of Pubmed and OVID databases was conducted since the inception to 01 April 2024. 24 articles were included from the USA, UK, South Africa, India, Japan, Finland, Canada, Turkey, Mexico, Thailand, Greece, Israel and Australia. A total of 1964 liver transplants were included. Median age was 50 months with 47.8% males. Data related to demographics, incidence, donor and recipient CMV serological classification (Donor (D) and Recipient (R)) and risk factors for CMV viremia/disease were extracted from the articles and subsequently analysed. This review was registered in Prospero: CRD42023477476

**Results:**

The Incidence of CMV viremia and disease in pediatric liver transplant patients in this review were 34% and 8.4% respectively, with statistically significant higher risk in the CMV D+/R+ category compared to other D/R serologic groups (P value < 0.05). , Additional risk factors identified for CMV viremia/disease included younger age, biliary atresia, high steroid doses, and rejection attacks.

**Conclusion:**

This meta-analysis highlights that CMV viremia and disease post-liver transplantation are significant health concerns in pediatric populations. Identifying high risk populations for targeted CMV monitoring and potentially preemptive therapy to mitigate CMV-associated morbidity and mortality is a critical cornerstone of pediatric transplant medicine. Further studies are warranted to explore additional risk factors and to refine prevention and treatment strategies for CMV in pediatric transplant recipients.

**Disclosures:**

All Authors: No reported disclosures

